# Comparison of Thoracic Epidural Analgesia and Thoracic Paravertebral Block Applications in the Treatment of Acute Pain After Thoracotomy in Geriatric Patients

**DOI:** 10.7759/cureus.18982

**Published:** 2021-10-22

**Authors:** Musa Zengin, Ali Alagoz

**Affiliations:** 1 Anesthesiology and Reanimation, University of Health Sciences, Ankara Atatürk Chest Diseases and Thoracic Surgery Training and Research Hospital, Ankara, TUR

**Keywords:** thoracotomy, thoracic paravertebral block, thoracic epidural analgesia, postoperative analgesia, geriatric patients

## Abstract

Background

Thoracic epidural analgesia (TEA) and thoracic paravertebral block (TPVB) are commonly used in geriatric patients for pain management after thoracotomy. In this study, we aimed to investigate the effect of TEA and TPVB on postoperative analgesia in geriatric patients who underwent thoracotomy.

Methodology

Postoperative analgesia follow-up files of patients over 65 years of age who underwent thoracotomy were analyzed retrospectively. Patient’s demographic data, diagnosis, type of surgery, postoperative 24-hour mean arterial pressure (MAP), heart rate, respiratory rate, peripheral oxygen saturation, static/dynamic visual analog scale (VAS) scores, need for additional analgesics, global pain assessment, and side effects such as nausea, vomiting, hypotension, bradycardia, and respiratory depression were examined. The patients were divided into two groups: those treated with TEA (Group 1) and those treated with TPVB (Group 2).

Results

There was no statistically significant difference between the groups in terms of demographic data (p > 0.05). MAP in the TEA group was statistically significantly lower than in the second and sixth-hour TPVB group (p = 0.008, p < 0.001). VAS static scores in the TEA group were statistically significantly lower at 30 minutes (p = 0.001), and at one, two, six, twelve, and twenty-four hours compared to the TPVB group (p < 0.001, except at 30 minutes). VAS dynamic scores were statistically significantly lower in the TEA group at 30 minutes, and at one, two, six, twelve, and twenty-four hours compared to the TPVB group (p < 0.001). There was no statistically significant difference between the groups in terms of nausea, vomiting, hypotension, and bradycardia (p > 0.05). The use of additional analgesics in the TEA group was statistically significantly lower than in the TPVB group (p < 0.001).

Conclusions

More effective postoperative analgesia results with stable hemodynamic conditions were observed in geriatric patients who underwent TEA for thoracotomy compared to TPVB. Regarding side effects, although there was a lower incidence in TPVB, this was not statistically significant when compared to TEA. TEA, as a component of the multimodal analgesia approach, can be accepted as a safe and effective method in the elderly patient group who underwent thoracotomy.

## Introduction

Thoracotomy, which is one of the most painful surgical incisions, causes a significant amount of trauma in the anatomical structures that are sensitive to pain. Consequently, severe acute pain occurs in the postoperative period [1,2]. Acute pain is an important factor that not only prolongs hospital stay but also increases postoperative morbidity. If not treated adequately, it can cause chronic pain that can last for months. This can prevent patients from returning to their normal activities for a long time [3].

Several analgesic methods such as thoracic epidural analgesia (TEA), thoracic paravertebral block (TPVB), plane block, intercostal nerve block, pleural block, as well as systemic and intrathecal analgesics have been suggested for thoracotomy pain [4,5]. Although TEA is the gold standard in the treatment of pain after thoracotomy, the use of other regional blocks has increased because TEA is more invasive and has various side effects (such as hypotension, nausea, urinary retention, and respiratory depression) [2,6-8]. Epidural hematoma, which is also one of the most frightening complications in patients undergoing TEA, may develop due to anticoagulant treatments, which are frequently used, especially in elderly patients [9]. In thoracic surgery, TPVB has become a widely preferred method as an alternative analgesic treatment in recent years due to the side effects of TEA [4,6-8].

The elderly may often encounter difficulties in pain management [10]. In the elderly, difficulty in expressing pain can become an obstacle to pain assessment. They may also think that it is a natural process for the pain to develop with age. Therefore, failure to report pain in elderly patients should not be interpreted as the absence of pain, and comprehensive approaches should be used for pain assessment [11]. The reasons for inadequate pain control by clinicians include lack of education, inadequate pain assessment, and reluctance to administer opioids, which may result in inadequate analgesic therapy [12,13]. When the pharmacokinetic and pharmacodynamic changes that occur with aging are added to this situation, serious problems can be encountered. In addition, multidrug use due to morbidity in elderly patients may lead to an increased incidence of adverse drug effects [10].

In this study, we aimed to investigate the effect of TEA and TPVB treatments on postoperative analgesia in geriatric patients who underwent thoracotomy. Our primary aim was to compare the pain levels and additional analgesia requirements in the first 24 hours postoperatively in geriatric patients. The secondary aim was to compare the side effects that may develop due to analgesia treatment and the satisfaction of patients with postoperative analgesia.

## Materials and methods

**Figure 1:**
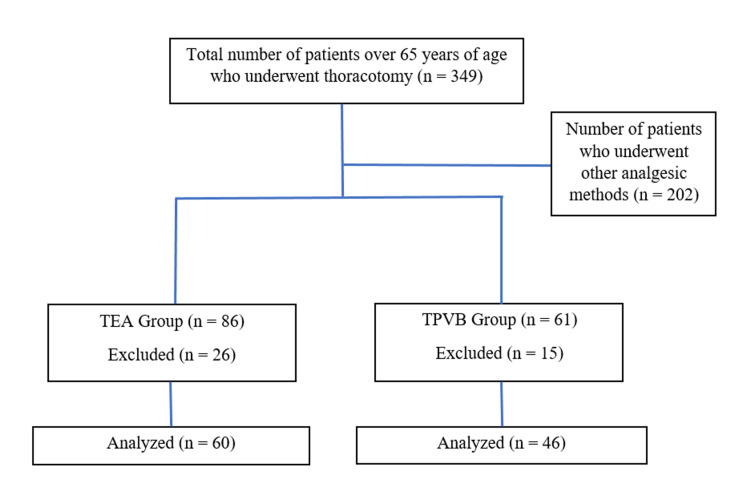
Flowchart of patients. TEA: thoracic epidural analgesia; TPVB: thoracic paravertebral block

After obtaining the approval of Ankara Keçiören Training and Research Hospital, Clinical Research Ethics Committee (2012-KEAK-15/2359), the data of the patients who underwent elective thoracotomy in our clinic were analyzed retrospectively. Thoracotomy was performed, TEA or TPVB treatment was applied for postoperative analgesia, and postoperative analgesia follow-up files of patients over 65 years of age were analyzed retrospectively. Patients’ age, gender, body mass index (BMI), American Society of Anesthesiologist (ASA) physical status, diagnosis, type of surgery, postoperative 24-hour mean arterial pressure (MAP), heart rate (HR), respiratory rate (RR), peripheral oxygen saturation (SpO_2_), static/dynamic visual analog scale (VAS) scores, need for additional analgesics, global pain assessment, and side effects such as nausea, vomiting, hypotension, bradycardia, and respiratory depression were examined. All patients were informed about the application and their consent was obtained. Those under the age of 65, did not undergo thoracotomy, were operated on in emergency conditions, had chronic pain before surgery, and constantly used analgesics or abused opioids were excluded from the study. The patients were divided into two groups: those treated with TEA (Group 1) and those treated with TPVB (Group 2) (Figure 1).

Thoracic epidural analgesia and thoracic paravertebral block protocols in study patients

TEA and TPVB procedures were explained in detail to the patients who were planned for thoracotomy. The patients were offered and received regional analgesia treatment. Intravenous (IV) patient-controlled analgesia (PCA) was administered to the patients who did not accept regional analgesia.

In the patients for whom TEA was planned, skin anesthesia was performed with 3 mL of 2% prilocaine after the skin was cleaned and covered following strict antisepsis rules in the sitting position. The epidural space was entered from the T5-T6 or T6-T7 vertebral spaces with an 18-gauge Tuohy needle using the median approach and the hanging drop method. Four cm of the catheter was left in the epidural space. To exclude vascular and intrathecal injection, a test dose (5 µg/mL (1:200,000) adrenaline and 3 mL 2% lidocaine) was administered through the epidural catheter, and the patients were placed in the supine position. The bilateral block was evaluated with a pinprick test. Intraoperatively, for epidural analgesia, 67.5 mL of bupivacaine, 201.5 mL of saline, and 10 mg/1 mL of morphine were mixed with a 270 mL elastomeric infusion pump. A concentration of 0.125% bupivacaine infusion was started and given epidurally for three days postoperatively, starting with an elastomeric pump at a rate of 4 mL/hour. In our study, we considered the first 24-hour follow-up to compare with TPVB.

For general anesthesia induction, 2 mg/kg propofol, 0.1 mg/kg vecuronium, and 1 µg/kg fentanyl were administered intravenously. Anesthesia was maintained in both groups by administering 50-100% O_2_-air mixture and 2% sevoflurane with remifentanil infusion (0.01-0.20 µg/kg/minute). If the continuation of neuromuscular blockade was necessary, IV 0.03 mg/kg vecuronium was administered. At the end of the surgery, 50 mg IV dexketoprofen and 100 mg IV tramadol were administered for analgesia, and 10 mg IV metoclopramide was administered as an antiemetic.

For the patients in the TPVB group, before general anesthesia, the insertion site was identified at 2.5 cm lateral of the spinous process at the level of T5-T6, and 3 mL of 2% prilocaine was administered for skin anesthesia. The nerve stimulator was set up at 0.1 ms, a frequency of 2 Hz, and a current of 2.5 mA. Subsequently, the 80-mm and 22-gauge (Stimuplex D Plus, Braun®, Melsungen, Germany) peripheral nerve stimulator needle was advanced. After the transverse process was felt with the needle, the needle was pulled back and directed 1 cm toward the upper side of the transverse process. The current was gradually reduced to 0.5 mA after determining that contractions were present in the intercostal muscles. Then, 20 mL of 0.5% bupivacaine was injected through the needle. The pinprick test was used to determine the block level after TPVB. Analgesia was maintained postoperatively in the surgical intensive care unit by IV PCA. According to our PCA protocol, 400 mg of tramadol was added to 100 mL of isotonic (0.9%) sodium chloride. Thus, a concentration of 4 mg/mL tramadol was obtained. It was then adjusted to 10 mg/hour basal infusion, 10 mg bolus, 20-minute lock-in time, and a four-hour limit of 100 mg. IV PCA treatment was applied for 24 hours.

For multimodal analgesia, all patients received paracetamol 1 g every eight hours, and 50 mg dexketoprofen IV was administered every 12 hours. Subsequently, 50 mg IV tramadol was given as an additional analgesic to patients with a VAS score of 4 and above. At the end of the pain management, the global pain rating was evaluated as 0 = poor, 1 = moderate, 2 = good, and 3 = excellent.

Statistical analyses

Data analyses were performed using SPSS for Windows, version 22.0 (IBM Corp., Armonk, NY, United States). Whether the distribution of continuous variables was normal was determined by the Kolmogorov Smirnov test. Levene test was used for the evaluation of homogeneity of variances. Unless specified otherwise, continuous data were described as mean ± standard deviation (SD) for normal distributions and as median (interquartile range) for skewed distributions. Categorical data were described as the number of cases and percentage (%). Statistical differences in normally distributed variables between two independent groups were compared by Student’s t-test. Mann-Whitney U test was applied for comparisons of non-normally distributed data. Categorical variables were compared using Pearson’s chi-square test or Fisher’s exact test. A P-value of <0.05 was considered significant for all statistical analyses.

## Results

The data of 106 patients over 65 years of age who underwent elective thoracotomy and received TEA or TPVB for postoperative analgesia were examined. When patients were compared regarding demographic data, diagnosis, and types of surgery, there was no statistically significant difference between the groups (p > 0.05) (Table 1).

**Table 1:** Demographic characteristics of patients, diagnosis, and operations. Continuous data are described as mean ± SD for normal distributions and median (interquartile range) for non-normal distributions. Categorical data are described as the number of cases (%). *Student’s t-test; ^β^Mann-Whitney U test; ^Φ^Pearson’s chi-square test or Fisher’s exact test; level of significance: p < 0.05. ASA: American Society of Anesthesiologists; BMI: body mass index; TEA: thoracic epidural analgesia; TPVB: thoracic paravertebral block

		TEA (n = 60)	TPVB (n = 46)	P-value
Gender	Male	52 (86.7%)	41 (89.1%)	0.702^Φ^
	Female	8 (13.3%)	5 (10.9%)	
Age, year [Median (interquartile range)]		67.0 (8.0)	68.0 (8.0)	0.076^β^
BMI, kg/m^2^ (Mean ± SD)		24.77 ± 4.19	23.67 ± 4.50	0.200*
ASA	ASA II	20 (33.3%)	18 (39.1%)	0.537^Φ^
	ASA III	40 (66.7%)	28 (60.9%)	
Diagnosis	Lung mass	50 (83.3%)	36 (78.3%)	0.487^Φ^
	Bronchiectasis	2 (3.3%)	-	
	Bullous lung disease	5 (8.3%)	7 (15.2%)	
	Hydatid cyst	3 (5%)	3 (6.5%)	
Surgery	Pneumonectomy	29 (48.3%)	24 (52.2%)	0.813^Φ^
	Lobectomy/Segmentectomy	28 (46.7%)	19 (41.3%)	
	Cystotomy/Capitonnage	3 (5%)	3 (6.5%)	

**Figure 2:**
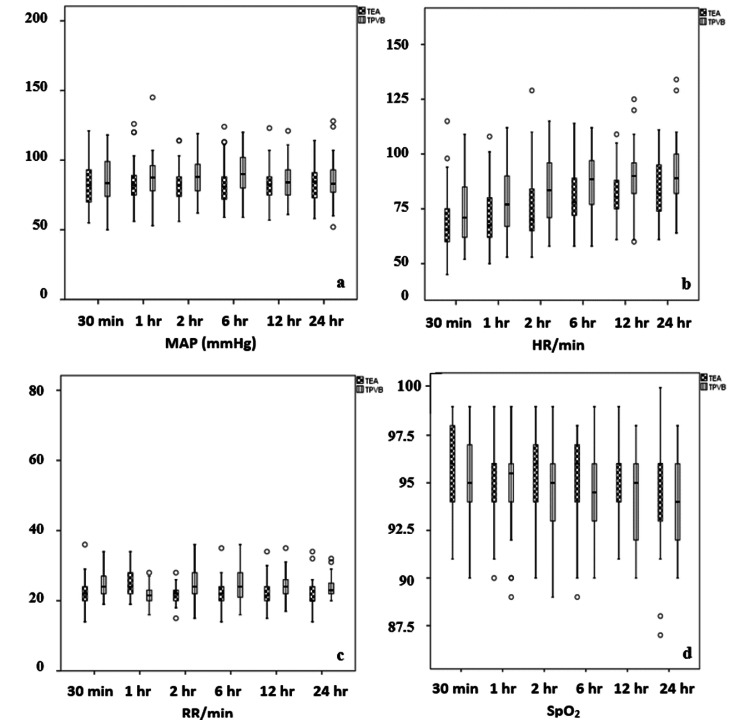
Boxplots for MAP, HR, RR, and SpO2. (a) Boxplot for MAP; (b) boxplot for HR; (c) boxplot for RR; (d) boxplot for SpO_2_. Data are expressed as median (horizontal bar), interquartile range (box), and maximum and minimum values (whiskers) for MAP, HR, RR, and SpO_2_. HR: heart rate; MAP: mean arterial pressure; RR: respiratory rate; SpO_2_: peripheral oxygen saturation

The MAP in the TEA group was statistically significantly lower than in the second and sixth-hour TPVB group (p = 0.008, p < 0.001) (Table 2; Figure 2a). The HR in the TEA group was statistically significantly lower than that after one hour (p = 0.014), two hours (p = 0.025), six hours (p = 0.049), twelve hours (p = 0.034), and twenty-four hours (p = 0.024) in the TPVB group (Table 2; Figure 2b). The RR in the TEA group was statistically significantly lower than that after thirty minutes (p = 0.001), one hour (p = 0.001), two hours (p < 0.001), six hours (p = 0.002), twelve hours (p < 0.001), and twenty-four hours (p < 0.001) in the TPVB group (Table 2; Figure 2c). There was no statistically significant difference between the groups concerning SpO_2_ (p > 0.05) (Table 2; Figure 2d).

**Table 2:** Comparison of groups in terms of MAP, HR, RR, and SpO2. Continuous data are described as mean ± SD for normal distributions and median (interquartile range) for non-normal distributions. *Student’s t-test; ^β^Mann-Whitney U test; level of significance: p < 0.05. MAP: mean arterial pressure; HR: heart rate, RR: respiratory rate, SpO_2_: peripheral oxygen saturation; TEA: thoracic epidural analgesia; TPVB: thoracic paravertebral block

	TEA (n = 60)	TPVB (n = 46)	P-value
MAP (Mean ± SD)			
30 minutes	84.38 ± 19.50	84.85 ± 16.49	0.764*
1 hour	82.50 ± 13.50	87.50 ± 18.00	0.140^β^
2 hours	83.00 ± 14.00	88.00 ± 19.00	0.008^β^
6 hours	80.82 ± 16.50	92.67 ± 18.84	<0.001*
12 hours	81.50 ± 14.00	84.00 ± 18.00	0.131^β^
24 hours	82.00 ± 15.00	83.00 ± 16.00	0.339^β^
HR (Mean ± SD)			
30 minutes	66.50 ± 14.00	71.00 ± 23.00	0.144^β^
1 hour	69.50 ± 18.50	77.00 ± 23.00	0.014^β^
2 hours	74.00 ± 21.00	83.50 ± 25.00	0.025^β^
6 hours	81.32 ± 14.33	86.59 ± 14.37	0.049*
12 hours	83.40 ± 13.92	88.91 ± 14.08	0.034*
24 hours	84.75 ± 13.36	91.02 ± 14.15	0.024*
RR (Mean ± SD)			
30 minutes	22.00 ± 4.00	24.00 ± 5.00	0.001^β^
1 hour	22.00 ± 4.00	24.00 ± 6.00	0.001^β^
2 hours	22.00 ± 4.00	24.00 ± 6.00	<0.001^β^
6 hours	22.00 ± 4.00	24.00 ± 7.00	0.002^β^
12 hours	21.00 ± 4.00	24.00 ± 4.00	<0.001^β^
24 hours	20.00 ± 4.50	23.00 ± 3.00	<0.001^β^
SpO_2 _(Mean ± SD)			
30 minutes	96.00 ± 4.00	95.00 ± 3.00	0.097^β^
1 hour	96.00 ± 1.50	95.50 ± 2.00	0.493^β^
2 hours	96.00 ± 3.00	95.00 ± 3.00	0.070^β^
6 hours	96.00 ± 3.00	94.50 ± 3.00	0.120^β^
12 hours	95.00 ± 2.00	95.00 ± 4.00	0.162^β^
24 hours	95.00 ± 3.50	94.00 ± 4.00	0.106^β^

VAS static scores in the TEA group were statistically significantly lower after thirty minutes (p = 0.001), one hour, two hours, six hours, twelve hours, and twenty-four hours compared to the TPVB group (p < 0.001, except at 30 minutes) (Table 3). VAS dynamic scores were statistically significantly lower in the TEA group after thirty minutes, one hour, two hours, six hours, twelve hours, and twenty-four hours compared to the TPVB group (p < 0.001) (Table 3).

**Table 3:** Comparison of groups in terms of static and dynamic VAS scores. ^β^Mann-Whitney U test; level of significance; p < 0.05. TEA: thoracic epidural analgesia; TPVB: thoracic paravertebral block; VAS: visual analog scale

	TEA (n = 60)	TPVB (n = 46)	P-value
	Median (Q_1_–Q_3_)	Median (Q_1_–Q_3_)	
VAS static			
30 minutes	3.00 (1.00)	4.00 (3.00)	0.001^β^
1 hour	3.00 (0.00)	4.00 (2.00)	<0.001^β^
2 hours	3.00 (1.00)	3.50 (1.00)	<0.001^β^
6 hours	2.00 (1.00)	3.00 (2.00)	<0.001^β^
12 hours	2.00 (1.00)	3.00 (1.00)	<0.001^β^
24 hours	1.00 (1.00)	2.50 (1.00)	<0.001^β^
VAS dynamic			
30 minutes	4.00 (1.00)	5.50 (3.00)	<0.001^β^
1 hour	4.00 (2.00)	5.00 (2.00)	<0.001^β^
2 hours	4.00 (2.00)	4.00 (1.00)	<0.001^β^
6 hours	3.00 (2.00)	4.00 (1.00)	<0.001^β ^
12 hours	2.00 (2.00)	4.00 (1.00)	<0.001^β^
24 hours	1.00 (1.00)	3.50 (1.00)	<0.001^β^

There was no statistically significant difference between the groups in terms of nausea, vomiting, hypotension, and bradycardia (p > 0.05). The use of additional analgesics in the TEA group was statistically significantly lower than that in the TPVB group (p < 0.001) (Table 4).

**Table 4:** Comparison of the groups in terms of side effects and additional analgesic requirements. Categorical data are described as the number of cases (%). ^Φ^Pearson’s chi-square test or Fisher’s exact test; level of significance: p < 0.05. TEA: thoracic epidural analgesia; TPVB: thoracic paravertebral block

	TEA (n = 60)	TPVB (n = 46)	P-value
	n (%)	n (%)	
Nausea	4 (6.7%)	2 (4.3%)	0.695^Φ^
Vomiting	1 (1.7%)	1 (2.2%)	0.999^Φ^
Hypotension	9 (15.0%)	2 (4.3%)	0.109^Φ^
Bradycardia	2 (3.3%)	-	0.504^Φ^
Additional analgesic requirements	11 (18.3%)	22 (47.8%)	<0.001^Φ^

There was a statistically significant difference between the groups in terms of global pain rating (p < 0.001). The satisfaction in the pain rating of the TEA group was higher than that of the TPVB group (Table 5).

**Table 5:** Comparison of groups in terms of the global pain rating. Categorical data are described as the number of cases (%). ^Φ^Pearson’s chi-square test or Fisher’s exact test; level of significance: p < 0.05. TEA: thoracic epidural analgesia; TPVB: thoracic paravertebral block

Global pain rating	TEA (n = 60)	TPVB (n = 46)	P-value
	n (%)	n (%)	
Poor	-	4 (8.7%)	<0.001^Φ^
Moderate	7 (11.7%)	20 (43.5%)	
Good	30 (50.0%)	22 (47.8%)	
Excellent	23 (38.3%)	-	

## Discussion

The results of the present study showed stable hemodynamic conditions and vital signs with effective postoperative analgesia in geriatric patients who underwent TEA. Concerning side effects, although there was a lower incidence with TPVB and IV infusion, it was not statistically significant when compared with patients who received TEA.

Evaluation and treatment of pain in elderly patients is very challenging [10]. One of the most important barriers to the difficulties in expressing pain is that elderly patients consider the pain to be a normal experience of old age [11]. On adding clinicians’ lack of training in pain control and inadequate pain assessment, analgesia management may be inadequate. Another important factor is the reluctance to administer opioids [12,13].

Increased sensitivity to opioids, especially due to pharmacodynamic and pharmacokinetic changes such as decreased hepatic metabolism and renal excretion, may occur in elderly patients. In addition, the use of multiple drugs due to morbidity in elderly patients may cause an increase in the incidence of side effects [10].

TEA or TPVB are widely used in pain management after thoracotomy for years [4,5]. TEA is crucial for reducing the stress response related to surgery and preventing postoperative complications. However, side effects such as hypotonia, urinary retention, and weakness in respiratory muscles may occur due to TEA administration in studies. In addition, neurological complications, epidural abscess, and hematoma related to the application, although rare, are feared complications [14]. TPVB may be an alternative to TEA due to its limited side effects and better safety margins, particularly among elderly patients who are administered anticoagulant therapy [15,16]. On the other hand, in a study by Wojtyś et al. [14] comparing TEA and TPVB, complication rates were observed to be similar in both groups. Similar results were found in our study in terms of complications.

One of the most important parameters in postoperative analgesic follow-up in elderly patients is a stable hemodynamic status and vital functions. Hypotension and bradycardia can be seen in TEA, especially due to the effect of the sympathetic block, which may increase morbidity and interrupt analgesic therapy [17]. In our study, a more stable hemodynamic response was observed in the TEA group in the postoperative period; no hemodynamic condition requiring ephedrine or atropine was encountered. In addition, the stable state observed in respiratory parameters suggests that TEA can be used effectively for postoperative analgesia in elderly patients.

Effective analgesia management after thoracotomy can reduce the early postoperative complications that may develop due to pain and limit the chronic post-thoracotomy pain that develops in the long term [18]. TEA and TPVB are considered among the most effective methods for the management of effective multimodal analgesia. Although several studies have been conducted in this field, we could not find any specific studies investigating the geriatric patient group. Different results were found in studies comparing TEA and TPVB. While Abd El-Hamid and Azab [19] found TPVB to be superior to TEA in thoracotomy, especially in pain control, Tamura et al. [2] observed that TEA provided better pain control than TPVB. In our study, similar to the findings of Tamura et al. [2], pain control was found to be superior in the TEA group.

The presence of comorbidities, multiple medications, and the pharmacokinetic and pharmacodynamic differences of the drugs used due to age and comorbidities, especially in elderly individuals, requires a comprehensive evaluation of dose adjustment [10]. Techniques that include regional analgesia as a component of multimodal analgesia can reduce the use and possible side effects of systemic drugs, especially opioids [20]. In this study, local anesthetic and opioid infusion were administered using an elastomeric infusion pump in the TEA group, and IV tramadol was added to the treatment to prevent possible inadequate treatment. In addition, multimodal analgesia with nonsteroidal anti-inflammatory drugs and paracetamol was administered to patients, and the synergistic effect of many drugs and methods in limited doses was used. In our view, this situation prevents the use of high-dose drugs to prevent pain and limits the side effects that may develop due to drugs in geriatric patients. Effective analgesia is provided in TPVB using the same multimodal analgesia technique. However, when static and dynamic VAS scores were compared with TPVB, it was observed that more effective analgesia was provided in TEA.

This study has some limitations. First, the study was retrospective and was conducted in a single center. Second, we recorded the patients’ pain scores only postoperatively within the first 24 hours. In this study, TPVB application was not performed under ultrasound guidance. However, the localization of the block site was determined using a nerve stimulator. In addition, a sufficient level of the block was confirmed by performing the pinprick test on the patients. Even though we evaluated acute postoperative pain, the follow-up of chronic post-thoracotomy pain that may develop can provide significant results in comparing TEA and TPVB.

## Conclusions

Effective postoperative analgesia results were obtained with TEA in geriatric patients who underwent thoracotomy. In addition, stable hemodynamic conditions and vital signs were achieved in patients. Although there was a lower incidence of TPVB in terms of side effects, it was not statistically significant when compared to patients receiving TEA. In addition, global patient satisfaction was better in patients who underwent TEA. As a component of the multimodal analgesia approach, TEA can provide effective analgesia in patients with an optimal dose adjustment by evaluating common comorbidities and long-term medications used in the geriatric patient group. In conclusion, it can be considered a safe and effective method in the elderly patient group who underwent thoracotomy. Large-scale prospective randomized studies on this subject will guide the safe use of TEA in geriatric patients.

## References

[1] Khelemsky Y, Noto CJ (2012). Preventing post-thoracotomy pain syndrome. Mt Sinai J Med.

[2] Tamura T, Mori S, Mori A, Ando M, Yokota S, Shibata Y, Nishiwaki K (2017). A randomized controlled trial comparing paravertebral block via the surgical field with thoracic epidural block using ropivacaine for post-thoracotomy pain relief. J Anesth.

[3] Khalil AE, Abdallah NM, Bashandy GM, Kaddah TA (2017). Ultrasound-guided serratus anterior plane block versus thoracic epidural analgesia for thoracotomy pain. J Cardiothorac Vasc Anesth.

[4] Davies RG, Myles PS, Graham JM (2006). A comparison of the analgesic efficacy and side-effects of paravertebral vs epidural blockade for thoracotomy--a systematic review and meta-analysis of randomized trials. Br J Anaesth.

[5] Joshi GP, Bonnet F, Shah R (2008). A systematic review of randomized trials evaluating regional techniques for postthoracotomy analgesia. Anesth Analg.

[6] Zengin M, Baldemir R, Ulger G, Sazak H, Alagoz A (2021). Postoperative analgesic efficacy of thoracic paravertebral block and erector spinae plane block combination in video-assisted thoracic surgery. Cureus.

[7] Barbera C, Milito P, Punturieri M, Asti E, Bonavina L (2017). Serratus anterior plane block for hybrid transthoracic esophagectomy: a pilot study. J Pain Res.

[8] Karmakar MK (2001). Thoracic paravertebral block. Anesthesiology.

[9] Burjorjee JE, Rooney R, Jaeger M (2018). Epidural hematoma following cessation of a direct oral anticoagulant: a case report. Reg Anesth Pain Med.

[10] Cavalieri TA (2007). Managing pain in geriatric patients. J Osteopath Med.

[11] Herr KA, Garand L (2001). Assessment and measurement of pain in older adults. Clin Geriatr Med.

[12] Gloth FM 3rd (2001). Pain management in older adults: prevention and treatment. J Am Geriatr Soc.

[13] Baldemir R, Akçaboy EY, Noyan Ö, Akçaboy ZN, Baydar M, Çelik Ş (2019). [An assessment of physicians attitudes toward opioid usage and opiophobia: results of a survey from a training and research hospital]. Agri.

[14] Wojtyś ME, Wąsikowski J, Wójcik N, Wójcik J, Wasilewski P, Lisowski P, Grodzki T (2019). Assessment of postoperative pain management and comparison of effectiveness of pain relief treatment involving paravertebral block and thoracic epidural analgesia in patients undergoing posterolateral thoracotomy. J Cardiothorac Surg.

[15] Mohta M, Verma P, Saxena AK, Sethi AK, Tyagi A, Girotra G (2009). Prospective, randomized comparison of continuous thoracic epidural and thoracic paravertebral infusion in patients with unilateral multiple fractured ribs--a pilot study. J Trauma.

[16] D'Ercole F, Arora H, Kumar PA (2018). Paravertebral block for thoracic surgery. J Cardiothorac Vasc Anesth.

[17] Koh JC, Song Y, Kim SY, Park S, Ko SH, Han DW (2017). Postoperative pain and patient-controlled epidural analgesia-related adverse effects in young and elderly patients: a retrospective analysis of 2,435 patients. J Pain Res.

[18] Ochroch EA, Gottschalk A (2005). Impact of acute pain and its management for thoracic surgical patients. Thorac Surg Clin.

[19] Abd El-Hamid AM, Azab AF (2016). Intraoperative haemodynamic stability and stress response to surgery in patients undergoing thoracotomy: comparison between ultrasound-assisted thoracic paravertebral and epidural block. Egypt J Cardiothorac Anesth.

[20] Nordquist D, Halaszynski TM (2014). Perioperative multimodal anesthesia using regional techniques in the aging surgical patient. Pain Res Treat.

